# Development of Lightweight Mortars Using Sustainable Low-Density Glass Aggregates from Secondary Raw Materials

**DOI:** 10.3390/ma16186281

**Published:** 2023-09-19

**Authors:** Maximina Romero, Isabel Padilla, José Luis García Calvo, Pedro Carballosa, Filipe Pedrosa, Aurora López-Delgado

**Affiliations:** Eduardo Torroja Institute for Construction Sciences, IETcc, CSIC, 28033 Madrid, Spain; isabel.padilla@ietcc.csic.es (I.P.); jolgac@ietcc.csic.es (J.L.G.C.); carballosa@ietcc.csic.es (P.C.); filipe.pedrosa@csic.es (F.P.); alopezdelgado@ietcc.csic.es (A.L.-D.)

**Keywords:** lightweight expanded glass aggregates, LEGAs, lightweight mortars, microstructure, compressive strength, flexural strength

## Abstract

In this study, different lightweight expanded glass aggregates (LEGAs) were produced from glass cullet and various carbonated wastes, through a thermal impact process. The effects of LEGA microstructure and morphology on both the adherence to the cement paste and the mechanical properties of mortars after 28 days of curing were studied. The properties of lightweight mortars made of either LEGAs or expanded clay aggregates were compared. The results demonstrated the feasibility of using LEGAs to produce glass lightweight aggregate mortar, with flexural and compressive strength values ranging from 5.5 to 8.2 MPa and from 28.1 to 47.6 MPa, respectively. The differences in mechanical properties were explained according to the microstructures of the fracture surfaces. Thus, arlite-type ceramic aggregates presented surface porosities that allowed mortar intrusion and the formation of an interconnected interface; although the surfaces of the vitreous aggregates were free from porosity due to their vitreous nature, the mortars obtained from different wastes presented compressive and flexural strengths in the range of lightweight mortars.

## 1. Introduction

The circular economy is a concept that aims to maintain the value of materials and resources in the market for as long as possible, while minimizing waste generation. The focus of the circular economy is the efficient use of resources, with attention on the reuse and recycling of waste, to turn our waste into raw materials. In this manner, waste from certain industrial processes becomes a resource for other processes. Within the framework of the circular economy, the European Commission recently launched two policy initiatives: the European Green Deal [1], which is a roadmap for a sustainable EU economy, and the Circular Economy Action Plan [2], which aims to transform the economy into a green, low-carbon economy in the future.

To achieve the European Green Deal, the Commission states that the focus of action should be on resource-intensive sectors, including the construction sector. The building and renovation of edifices requires considerable amounts of energy and mineral resources (sand, gravel, clay, water, etc.). Construction materials are one of the most suitable options for the reuse of waste. On the one hand, their manufacture requires the consumption of high volumes of natural resources. Thus, in 2022, world cement production was estimated at 4.1 billion tons [3], whereas the total global production volume of ceramic tiles amounted to 18.34 billion square meters [4]. On the other hand, building materials have heterogeneous compositions and microstructures; therefore, they tolerate compositional variations in waste from different campaigns. In recent decades, several studies have focused on the reuse of waste as a raw material in the manufacture of different construction materials, such as various types of clinker [5], cement [6,7,8], mortars [9], concretes [10,11,12,13], geopolymers [14,15], clay bricks [16,17,18,19,20], ceramic tiles [21,22], and roof tiles [23,24].

The use of waste in designing lightweight mortars and concretes has also been evaluated. Lightweight mortars (or concrete) can be defined as any type of mortar with an oven-dry density less than 2000 kg/m^3^ [25]. These mortars have several advantages, such as their use in non-structural applications due to their reduced weight, thermal insulation, and acoustic performance [26]. In fact, lightweight mortars and concretes yield significant reductions in the amount of material used, element size, construction time, handling and transport costs, and energy used [25,27]. Accordingly, the use of these special materials has progressively increased, and they are used in structural applications and even in the development of high-performance concretes [28].

In the manufacture of lightweight mortars, the use of different low-density aggregates, such as natural aggregates (pumice, perlite, or vermiculite) [29,30,31] and expanded clay [32], has been documented. Furthermore, within the framework of the circular economy, in recent years various research projects have been carried out to manufacture lightweight mortars with incorporations of different types of waste, such as fly ash aggregates [31,33,34], biochar [35,36], sludge from municipal sewage treatment plants [37], poly(terephtalate ethylene) (PET) flakes from recycled packages [38], and wood processing by-products [39].

One important waste stream is the glass cullet from the manufacture and consumption of glass packaging. Worldwide, the annual production of container glass is 95 million tonnes. In 2019, EU Member States produced 18.7 million tonnes of packaging glass waste. As glass is an inert, durable material that does not cause degradation reactions, considerable research has been carried out in recent years to replace fine natural aggregates in mortars and concretes with crushed glass waste [40,41,42,43]. The results of these studies conclude that the incorporation of glass aggregates improved the performance levels of mortars with respect to the deterioration from expansive salts or freeze/thaw cycles, showing superior acid resistance levels and a better behavior under certain high temperature conditions. Crushed glass waste has been reported as a fine aggregate in the production of geopolymer mortars, providing similar reaction products to metakaolin and reducing the alkali–silica reaction [44,45,46,47,48].

Another method for reusing container waste glass is through its transformation into lightweight expanded glass aggregates, which can be used in the manufacture of lightweight mortars. Lightweight glass aggregates have a low density because of their cellular structure and high compressive strength [49]. In addition, these materials combine other characteristics of glass, such as chemical resistance and durability. The manufacture of these vitreous aggregates from container glass cullet has a beneficial environmental impact, as it is estimated that recycling one tonne of waste glass saves approximately 1.2 tonnes of raw materials, resulting in a reduction of 0.67 tonnes of CO_2_ emissions in the production of glass lightweight aggregates [50]. Notably, to the authors’ knowledge, the literature lacks information on the manufacture and characterization of lightweight mortars containing glass lightweight aggregates. In this respect, only one recent study reported the influences of different glass aggregates, including expanded glass, on the shrinkage and expansion of cement mortar [51]. In this research, the authors used commercial expanded glass with sizes ranging from 40 to 125 µm as a replacement for sand and concluded that the addition of expanded glass aggregate to cementitious mortar is beneficial if the type of aggregate and the amount are properly chosen. However, this very low particle size increases the density and cannot be easily used in many of the conventional applications of lightweight mortars, without its combination with aggregates of a higher particle size.

Therefore, the aim of this study was to determine the feasibility of using glass aggregates produced from mixtures totally composed of wastes (glass cullet as base materials and carbonated wastes as additives) in the manufacture of lightweight mortars and to evaluate their suitability for use in the development of lightweight mortars. The performance levels and microstructural properties of the resulting mortars were evaluated.

## 2. Materials and Methods

### 2.1. Lightweight Expanded Glass Aggregate Manufacture

Lightweight expanded glass aggregates (LEGAs) were entirely manufactured from secondary raw materials. Thus, a white container glass cullet (VERALLIA S. A, Spain) was used as the base component, and different carbonated wastes were used as expansion agents. Three residues from magnesite mining (Magnesitas de Navarra, Spain)—carbonate F (CF), carbonate PC8 (PC8), and flotation tailing (FT)—comprising dolomite (CaMg(CO_3_)_2_), magnesite (MgCO_3_), periclase (MgO), and quartz (SiO_2_) in different proportions were used as foaming agents. In addition, mussel shell (MS), composed mainly of aragonite and calcite (CaCO_3_), was used to induce glass cullet foaming. LEGAs were manufactured from different wastes with a particle sizes below 1 mm. When necessary, the residues were subjected to conditioning steps, such as oven drying at 120 °C for at least 24 h, grinding in a planetary mill (RETSCH PM 100), and sieving.

**Figure 1:**
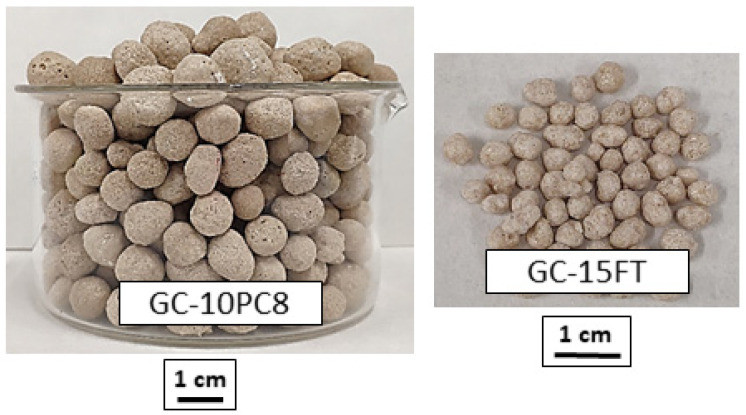
Macroscopic appearance of the manufactured lightweight expanded glass aggregates.

LEGAs were manufactured from mixtures of the container glass cullet (GC) and each foaming agent. The tested compositions were GC-5MS, GC-15FT, GC-10CF, and GC-10PC8, where the number preceding the abbreviation of the foaming additive indicated its percentage in the mixture. The optimization of the composition and the processing conditions were comprehensively described in a previous study [49]. For the manufacture of glass aggregates, 500 g of each composition was mixed and homogenized in a planetary ball mill (TURBULA) for 15 min. Subsequently, the mixtures were pelletized into 1–2 mm granules, which were oven-dried at 105 °C for 24 h. For expansion, the green pellets were placed in an electric furnace preheated to 800 °C and maintained for 15 min. Subsequently, the pellets were extracted from the furnace and cooled in air. After expansion, the aggregate particle size was in the 2–4 mm range. Figure 1 shows the macroscopic appearance of the aggregates manufactured.

To evaluate the effects of the morphologies of the LEGAs on the adherence to the cement paste (the cement paste–aggregate interface) and consequently on the mechanical properties of the lightweight mortars, two morphologies (rounded and angular) were considered for GC-15FT. Angular fragments (GC-10CF-a) were prepared by crushing and sieving large aggregates.

### 2.2. Lightweight Mortar Manufacture

Six different lightweight mortars were designed. In five of them, LEGAs were used as lightweight aggregates. In addition, a reference lightweight mortar manufactured with a commercial lightweight aggregate (arlite) was used for comparison reasons. The arlite had the same particle size as the LEGA (2–4 mm). The mortar compositions are shown in Table 1. To obtain comparable results, the *w*/*c* ratio, the cement content, and the volume of the different raw materials were maintained in all cases. A siliceous sand according to EN 196-1 [52] (0–2 mm size) was used in all the mortars, to be combined with the lightweight aggregates. The lightweight aggregates were water saturated before the fabrication of the mortar samples, to guarantee the same effective *w*/*c* (0.45) in all cases. Moreover, all the lightweight mortars had similar fluid consistencies in the fresh state. Three 4 × 4 × 16 cm prismatic samples were fabricated for each of the mortar compositions, to evaluate their mechanical properties after 28 days of curing (98% RH and 20 °C).

### 2.3. Material Characterisation

The densities and absorptions of the fabricated glass lightweight aggregates and those of the arlite used were measured according to EN 1097-6 [53], using the pycnometer method. Particle density and water absorption was calculated from the ratio of mass to volume of the aggregates, water, and pycnometer. The mass was determined by weighing the test aggregate portion in saturated and surface-dried conditions and again in the oven-dried condition.

A microstructural study of the glass lightweight aggregates was carried out using field emission scanning electron microscopy (FESEM) in a Hitachi Model S-4800 microscope. The observations were performed on cross-sections of expanded glass aggregates and on fresh fracture surfaces of lightweight mortars. In all cases, the samples were coated with a thin carbon layer for easier observation.

The apparent densities of the fresh mortars were measured according to EN 1015-6 [54] using the pressure method, where an air meter (modified Washington type) was used. The flexural and compressive strengths of the fabricated mortars were evaluated after 28 days of standard curing according to EN 196-1 (98% RH and 20 °C) [52]. Three identical specimens were tested in each case; thus, three flexural strength measurements and six compressive strength measurements were made for each mortar type.

## 3. Results and Discussion

### 3.1. Lightweight Expanded Glass Aggregates

The particle densities of the lightweight aggregates are shown in Table 2. According to the standards, an aggregate is considered a light aggregate if its particle density is lower than 2.00 g/cm^3^ according to EN 13055-1 [55]. In accordance with Table 2, all the studied glass aggregates were lightweight aggregates, although a lower density was obtained in the commercial aggregate. Although all the aggregates were derived from the same base glass, their densities were quite dissimilar. The lowest density of glass aggregate was obtained in the GC-15FT sample; notably, the density of the angular aggregate (GC-15FT-a) was slightly lower. GC-10PC8 showed the highest particle density. As explained below, the differences observed in the density values of the different aggregates were the result of the differences between the decomposition temperatures of the carbonates present in the additives and the glass transition temperature (Tg) of the base glass.

**Figure 2:**
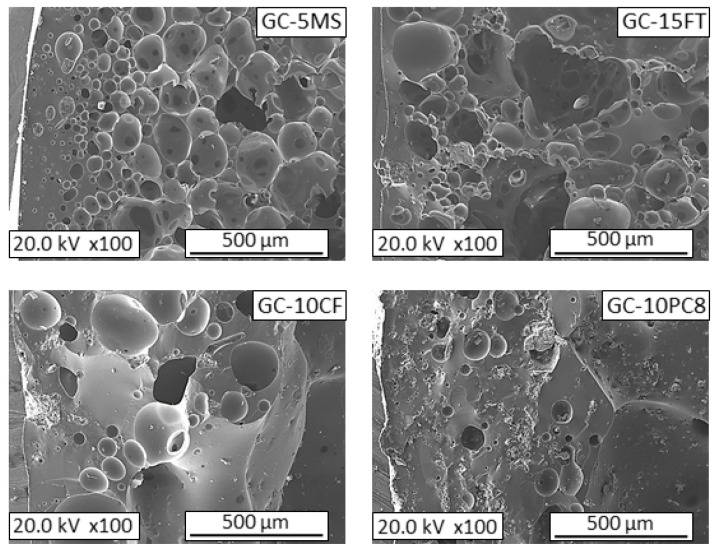
FESEM observations of the microstructures of glass aggregates prepared with different foaming additives: GC-5MS, GC-15FT, GC-10CF, and GC-10PC8.

Figure 2 shows FESEM images of the microstructure observed in cross-section samples of the expanded glass aggregates. Similarly to the density values, the microstructures of the aggregates differed to some extent. The GC-5MS aggregate presented the most homogeneous microstructure, composed of a dense shell containing small isolated spherical bubbles; the sizes increased towards the interior of the aggregate, while the interconnection between the bubbles increased. Considering that the mean density of glass is 2500 kg/m^3^, the porosities of the GC-5MS aggregates were estimated at approximately 50%. The porosities of the GC-15FT aggregates were slightly higher (60%); however, they showed more heterogeneous microstructures, with irregularly shaped bubbles of varying sizes, with large voids that exceed 500 µm. Moreover, the GC-10CF and GC-10PC8 aggregates were the densest, with porosity values of 20% and 8%, respectively.

The observed differences in the density and porosity values of the different aggregates were the result of the different mineralogical compositions of the additives used as foaming agents and, more specifically, the gaps between the decomposition temperatures of the carbonates present in the additives and the glass transition temperature (Tg) of the base glass (approximately 610 °C) [49]. In these materials, the expansion of the aggregate was caused by the release of CO_2_ gas because of the decomposition of the carbonates in the additives. Depending on the temperature at which decomposition occurs, the released gases flow through a matrix of different plasticity, restricting the degree of expansion of the final aggregate. Overall, the microstructures of the green pellets consisted of glass grains with irregular morphologies and voids between particles. During heating, the microstructure remains unchanged until the glass transition temperature is reached, at which point the glass particles change from a brittle glassy state to a flexible rubbery state [56]. As the temperature increases, particle rearrangement occurs, while the initial open porosity decreases and becomes closed porosity [57]. The decarbonation processes of CaCO_3_ in MS and FT start at 750 and 800 °C, respectively [49], which are far above the Tg of the base glass. Gases are discharged into a viscous sintered matrix with reduced open porosity, becoming trapped, leading to matrix blowing and resulting in high porosity aggregates. Conversely, the decarbonation of MgCO_3_ in CF and PC8 additives starts below 600 °C [49], which is before the Tg of the base glass is reached. In such cases, the released gases escape through the open porosity before the sintering of the glass particles progresses, resulting in aggregates with reduced porosity.

### 3.2. Lightweight Mortar Characterisation

Table 3 collects the densities in the fresh state of the fabricated mortars and the mechanical properties after 28 days of curing. 

The flexural strength value refers to the mean value of three measurements; the compressive strength value refers to the mean value of six measurements. As expected, the density obtained was strongly related to the particle densities of the lightweight aggregates used. The densities of GC-5MS, GC-15FT, and GC-15FT-a were very similar to those obtained in the REF sample, while GC-10CF and GC-10PC8 had densities above 2 g/cm^3^. This aspect was manifested in the compressive strength results obtained. While GC-5MS, GC-15FT, and GC-15FT-a exhibited values of compressive strength similar to those obtained when using arlite, the compressive strengths of GC-10CF and GC-10PC8 were higher. The increase in the compressive strength with respect to the reference sample was approximately 50% in GC-10CF and 37% in GC-10PC8, while the increase in the fresh density was 15% and 14%, respectively. Thus, apart from their higher densities, both glass aggregates showed good properties for obtaining high mechanical properties. Although the densities of both mortars (GC-10CF and GC-10PC8) did not accurately classify them as lightweight mortars, the equilibrium density according to the ASTM C567 standard [58] was under 2000 kg/m^3^, thus confirming that they were lightweight mortars. Therefore, it is reasonable to conclude that both glass aggregates allow the design of lightweight mortars with good mechanical properties. In any case, possible combinations of these glass aggregates with those of a lower density (or even with arlite or another commercial lightweight aggregate) should be tested, in order to obtain mortars with a lower density and higher mechanical properties. Moreover, mortars with higher lightweight aggregate content than the ones tested in the present study should be also evaluated. In the case of the GC-5MS, GC-15FT, and GC-15FT-a glass aggregates, the resulting mortars were classified as lightweight, and their properties were very similar to those obtained with arlite of a similar particle size. The morphology of the GC-15FT aggregate (rounded or angular) slightly modified the strength properties of the resulting mortars. This factor, particle morphology in addition to density, could explain the different mechanical properties presented by mixtures GC-10PC8 and GC-10CF. The GC-10PC8 sample presented a particular shape, in which an angular morphology predominated compared to the more spherical one detected in the case of the GC-10CF sample. These morphologies and their interaction with the interfacial zone could have directly influenced the mechanical behavior observed in compression and in flexion between these two mixtures. However, further studies are needed to improve the understanding of this effect.

**Figure 3:**
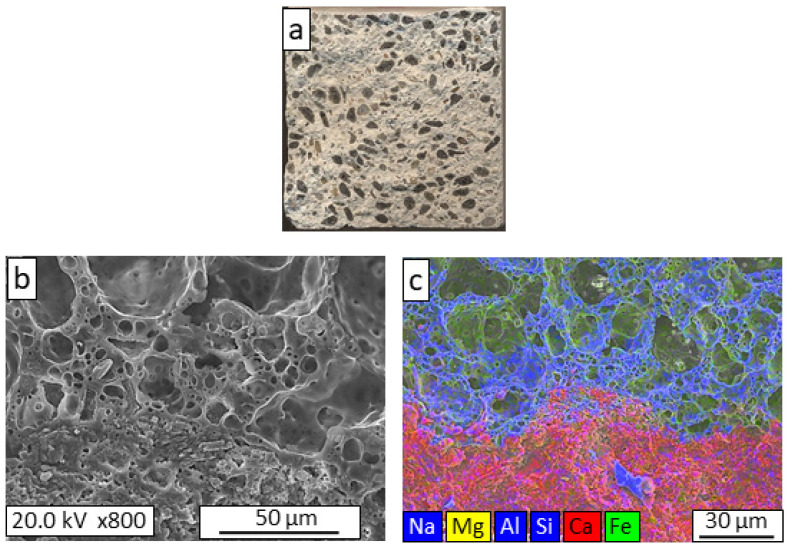
Macrostructural appearance (**a**); FESEM observation (**b**); and elemental mapping (**c**) of the fracture surface of REF lightweight mortar.

**Figure 4:**
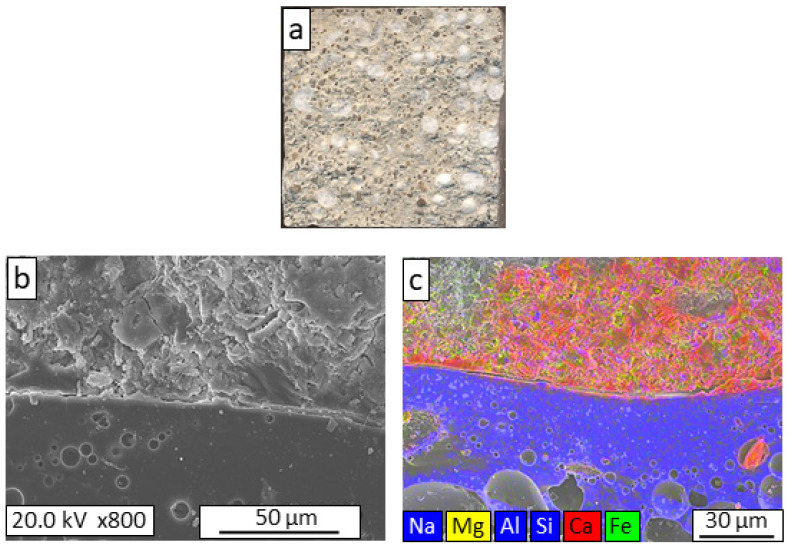
Macrostructural appearance (**a**); FESEM observation (**b**); and elemental mapping (**c**) of the fracture surface of GC-5MS lightweight mortar.

**Figure 5:**
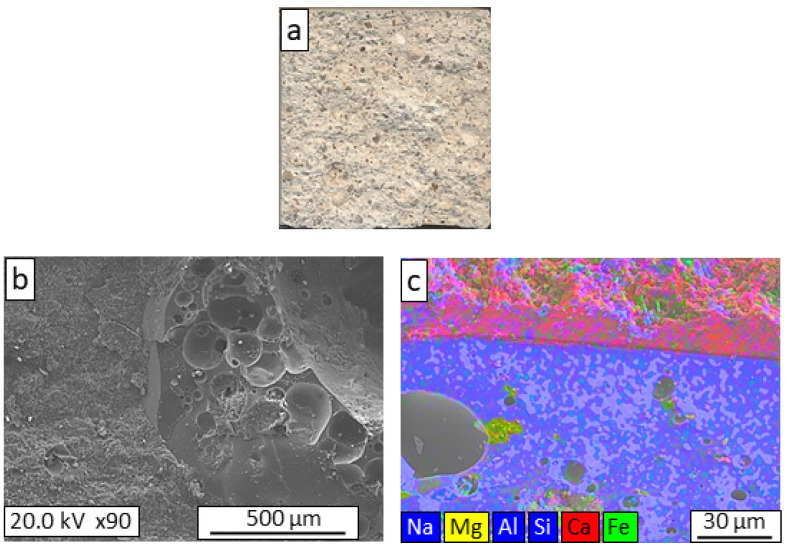
Macrostructural appearance (**a**); FESEM observation (**b**); and elemental mapping (**c**) of the fracture surface of GC-15FT lightweight mortar.

**Figure 6:**
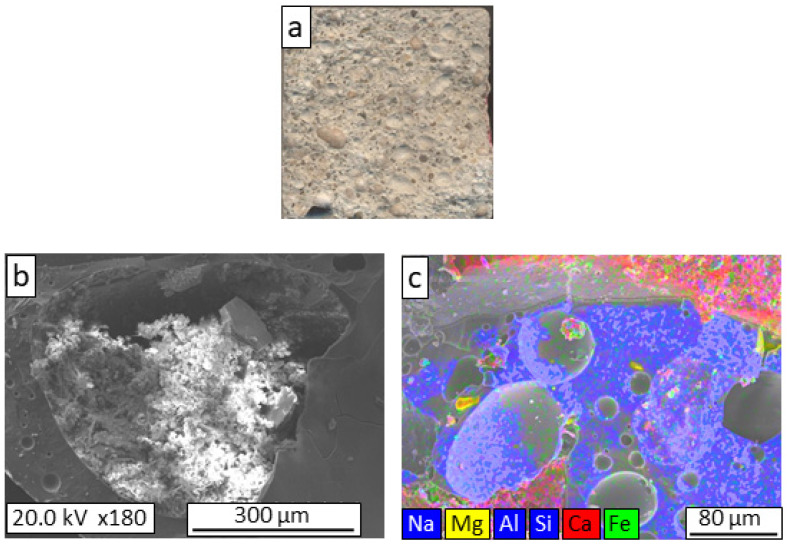
Macrostructural appearance (**a**); FESEM observation (**b**); and elemental mapping (**c**) of the fracture surface of GC-10CF lightweight mortar.

**Figure 7:**
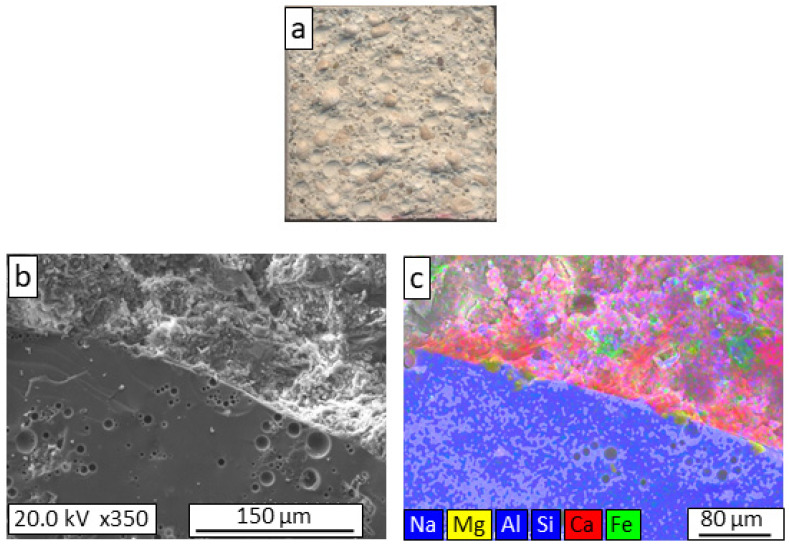
Macrostructural appearance (**a**); FESEM observation (**b**); and elemental mapping (**c**) of the fracture surface of GC-10PC8 lightweight mortar.

For a better understanding of the observed variations in the mechanical properties of the lightweight mortars, textural and microstructural studies of their failure areas were carried out. Figure 3, Figure 4, Figure 5, Figure 6 and Figure 7 show the macrostructural appearances and FESEM observations of the fracture surfaces. Macroscopically, the fracture zone of the REF mortar (Figure 3a) generally showed intragranular fracture of the arlite, which indicated good adherence between the lightweight aggregate and the mortar. The microstructure image (Figure 3b) shows the existence of an undefined boundary between the arlite particles and the mortar, indicating a close bond between the two phases; this phenomenon is clearly seen in the mapping image (Figure 3c), where an interconnected aggregate–mortar interface can be observed.

In the fracture zone of the GC-5MS lightweight mortar prepared with MS aggregate (Figure 4a), fractured aggregate particles are observed; some whole particles and voids were caused by the detachment of aggregate particles during the mechanical test. This observation indicates that the aggregate–mortar interface was weaker than that of the reference mortar. In the SEM observations (Figure 4b,c), there is no clear evidence of a region where the mortar and aggregate are mixed, which is attributed to the different surface characteristics of the reference aggregate (ceramic aggregate) and the aggregates under study (glass aggregates). The arlite-type ceramic aggregates had surface porosities that allowed the intrusion of mortar and the formation of an interconnected interface. In contrast, the surfaces of the glass aggregates were free of porosity. Despite this result, the mortar manufactured with the GC-5MS aggregate (M-GC-5MS) presented mechanical properties similar to those of the reference mortar.

In the fracture zone of the M-GC-15FT mortar (Figure 5a), a greater extent of intergranular fracture of the aggregate particles was observed, as indicated by the presence of whole particles and voids produced by the detachment of entire particles. This microstructural feature confirmed that the flexural strength of the mortar was lower than those of the reference and GC-5MS mortars. As with the GC-5MS mortar, no mixing zone was visible between the mortar and the aggregates (Figure 5b,c). However, the compressive strength was lower than that of the previous mortars, which was attributed to the lower thickness of the dense shell of the GC-15FT aggregate than that of the GC-5MS (Figure 2).

In the GC-10CF mortar (Figure 6a), intergranular fracturing was widespread and much more significant than the intragranular fracturing. However, in the SEM images (Figure 6b,c), it can be observed that the pores existing at the surface of the glass aggregate are filled with the mortar paste. With the higher density of the aggregate, this fact explains the higher increase in compressive strength (50% higher) relative to the reference material and to the other mortars produced with glass lightweight aggregates.

The macrostructural appearance of the fracture area of GC-10PC8 (Figure 7a) is similar to that of the GC-10CF mortar. However, in this case, no penetration of the mortar paste into the surface pores of the aggregate is observed (Figure 7b,c); this glass aggregate was the densest of the aggregates studied and presented the smallest volume of pores close to the surface. The difference in compressive strength relative to the reference mortar was 37% higher, mainly due to the relatively high density of the aggregate.

## 4. Conclusions

Lightweight mortars with technical properties similar to those of mortar prepared from commercial lightweight aggregate were manufactured using synthetic LEGAs entirely prepared using different wastes (glass cullet and carbonated wastes) as raw materials. The principal characteristics of these lightweight mortars were as follows: fresh state density of 1.81–2.01 g/cm^3^, flexural strength of 5.5–8.2 MPa, and compressive strength of 28.1–47.6 MPa, depending on the lightweight aggregate used. These findings could serve as important contributions to the concepts of the circular economy and environmental sustainability, because of the preservation of natural resources and the use of waste as a secondary raw material. In addition, these findings could contribute to the 2030 Agenda for Sustainable Development and toward the recommendations of the European Platform for Recycled Aggregates.

## Figures and Tables

**Table 1:** Mortar compositions (expressed in grams).

Content (g)	REF	GC-5MS	GC-15FT	GC-15FT-a	GC-10CF	GC-10PC8
Cement	550	550	550	550	550	550
Water	250	250	250	250	250	250
Siliceous sand	825	825	825	825	825	825
Arlite	255	-	-	-	-	-
GC-5MS	-	395	-	-	-	-
GC-15FT	-	-	330	-	-	-
GC-115FT-a	-	-	-	315	-	-
GC-10CF	-	-	-	-	550	-
GC-10PC8	-	-	-	-	-	690

**Table 2:** Particle densities of arlite and the synthetic glass aggregates.

Aggregate	Arlite	GC-5MS	GC-15FT	GC-15FT-a	GC-10CF	GC-10PC8
Dry density (g/cm^3^)	0.77	1.21	1.00	0.95	1.67	2.09
Water absorption (%)	1.7	4.4	18.5	24.9	3.6	2.8
Saturated surface dry density (g/cm^3^)	1.79	1.33	1.46	1.55	1.84	2.29

**Table 3:** Properties of the lightweight mortars.

Property	REF	GC-5MS	GC-15FT	GC-15FT-a	GC-10CF	GC-10PC8
Fresh state density (g/cm^3^)	1.81	1.92	1.91	1.87	2.08	2.07
Flexural strength at 28 days (MPa)	7.1	7.1	6.6	5.5	7.3	8.2
Compressive strength at 28 days (MPa)	31.7	31.4	28.1	29.7	47.6	43.5

## Data Availability

Not applicable.
